# A randomized, double-blind, placebo-controlled, phase II dose-finding study of the short acting β_1_-blocker, landiolol hydrochloride, in patients with suspected ischemic cardiac disease

**DOI:** 10.1007/s10554-013-0253-3

**Published:** 2013-06-20

**Authors:** Masahiro Jinzaki, Masaharu Hirano, Kazuhiro Hara, Takahiko Suzuki, Akira Yamashina, Yuji Ikari, Misako Iino, Takuhiro Yamaguchi, Sachio Kuribayashi

**Affiliations:** 1 Department of Diagnostic Radiology, Keio University School of Medicine, 35 Shinanomachi, Shinjuku-ku, Tokyo, 160-8582 Japan; 2 Department of Cardiology, Tokyo Medical University Hospital, Tokyo, Japan; 3 Department of Cardiology, Mitsui Memorial Hospital, Tokyo, Japan; 4 Department of Cardiology, Toyohashi Heart Center, Toyohashi, Japan; 5 Department of Cardiology, Tokai University School of Medicine, Tokyo, Japan; 6 Department of Radiology, Tokai University School of Medicine, Tokyo, Japan; 7 Division of Biostatistics, Tohoku University Graduate School of Medicine, Tohoku, Japan

**Keywords:** Computed tomography, Beta-blocker, Safety, Heart rate, Coronary artery

## Abstract

The purpose of this study was to compare the safety and efficacy of the short-acting β_1_-receptor blocker, landiolol hydrochloride (0.06 and 0.125-mg/kg), to placebo during coronary computed tomography angiography (CTA) in a phase 2 dose-finding study. A total of 183 patients suspected of having ischemic cardiac disease and scheduled to undergo an invasive coronary angiography were randomized to groups treated with landiolol hydrochloride (0.06 or 0.125-mg/kg) or placebo. The heart rate, safety, and the performance of coronary diagnosis using landiolol hydrochloride were evaluated in a multicenter, double-blind, randomized, parallel study. The patients’ heart rates during the coronary CTA were 67.6 ± 8.7 and 62.6 ± 7.8 beats/min in the 0.06 and 0.125-mg/kg landiolol hydrochloride groups, respectively, both of which were significantly lower than the heat rate of 73.7 ± 11.8 beats/min in the placebo group (*P* = 0.003 and *P* < 0.001, respectively). No adverse events or reactions occurred at an incidence of 5 % or greater, confirming the safety of landiolol hydrochloride. The proportion of correctly classified patients was significantly higher in the 0.125-mg/kg landiolol hydrochloride group than in the placebo group (73.6 vs. 50.0 %). Landiolol hydrochloride at doses of 0.06 and 0.125-mg/kg significantly decreased the heart rate compared with a placebo. The present findings suggest that landiolol hydrochloride is safe and useful at a dose of 0.125-mg/kg to improve coronary diagnostic performance during coronary CTA.

## Introduction

Coronary computed tomography angiography (CTA) is a non-invasive method for diagnosing the presence and extent of coronary artery stenosis [1, 2]. Single and multicenter studies have reported coronary CTA to be useful, and to have a very high negative predictive value [3–5]. However, poor image quality during CTA has been reported in patients with a high heart rate, necessitating the administration of a β-receptor blocker to decrease the heart rate and thus improve image quality by increasing the relative time resolution during coronary CTA [1, 2]. Many clinical studies have reported the administration of β-receptor blockers to lower the heart rate during coronary CTA [3–6].

Recently, high diagnostic capability without the use of β-receptor blockers has been reported using dual source CT, which shortens the imaging time and improves the time resolution [7]. However, these results were obtained using updated facilities. Common CT equipment presently in use still require the use of β-receptor blockers. Also, while the reduction of radiation exposure is a matter of serious concern in cardiac CT imaging, many techniques [such as electrocardiography (ECG)-dose modulation or “step and shoot” scans] that have been developed to address this problem can only be used at low heart rates [8–12]. Furthermore, new techniques, such as perfusion CT and dual energy CT, also require a low heart rate to maintain image quality [13–16]. Thus, the need for β-receptor blockers continues to be quite high in actual clinical practice.

The oral β-receptor blockers that have been widely used in previous studies require more than an hour to produce an adequate effect. Consequently, short-acting β-receptor blockers have been desired for better testing efficacy. In this study, we evaluated the usefulness and safety of a short-acting β-receptor blocker during coronary CTA using a placebo-controlled, double-blind study performed with a 64-row CT scanner.

## Materials and methods

The present study was a multicenter, double-blind, randomized, parallel study conducted at 18 study centers in Japan (http://clinicaltrials.gov/ct2/show/NCT00560209). The study period was from December 2007 to September 2008. This study was approved by the Institutional Review Boards (IRBs) of all of the study centers was conducted in accordance with the ethical principles that have their origins in the Declaration of Helsinki, and was in compliance with the standards pursuant to Article14, Paragraph 3 and Article 80-2 of the Pharmaceutical Affairs Law and the Ordinance on Standards for the Implementation of Clinical Studies on Drugs (GCP) (MHW Ordinance No. 28). Investigators or subinvestigators handed a consent form, approved by the IRB of each study center, to each of the participating patients before the study was started, carefully explained the study, and obtained informed consent from each patient.

Prior to coronary CTA, patients aged 20 years or over who were suspected of having ischemic cardiac disease based on inquiry regarding symptoms and tests including a standard 12-lead ECG examination, chest X-ray, or echocardiography, and who were scheduled to undergo coronary angiography (CAG) with cardiac catheterization following coronary CTA were selected if they met either of the following conditions: (1) chest pain with positive findings on exercise (ECG), or (2) positive findings on myocardial blood flow imaging or echocardiography. Additionally, their heart rate could not be less than 70 beats/min or greater than 90 beats/min on admission to the CT room and immediately before the administration of nitrate medication. Based on the efficacy data in a previous study of this drug, the mean percent change of heart rate from baseline was estimated to be approximately 10–27 % [17, 18]. Thus, in cases where the target heart rate is less than 65 bpm for coronary CTA, subjects with heart rates over 90 bpm will not be expected to achieve the target heart rate through administration of this study drug.

Patients were excluded from the present study if they had a cardiac pacemaker, defibrillator, or both implanted; had undergone coronary-artery bypass surgery; had atrial fibrillation or extrasystole; had a systolic blood pressure less than 110 mmHg before coronary CTA; or if they had been scheduled for interventional therapy between the coronary CTA and the CAG. The use of the following drugs was prohibited: β-receptor blockers, calcium channel blockers, antiarrhythmic agents, sympathomimetic agents, and biguanide antidiabetic agents. The following patients were also excluded from the present study: those contraindicated for the use of β-receptor blockers or nonionic contrast media, and those who were pregnant, lactating, possibly pregnant, or desiring to become pregnant during the study period.

Based on the previous Phase-II study (not published) where we explored and confirmed the efficacy and safety of 0.125, 0.25, and 0.5 mg/kg of this drug, we decided to run a comparison test of 0.125 and 0.06 mg/kg. Eligible patients were randomized (Permuted-block randomization) to groups treated with 0.06 or 0.125 mg/kg of landiolol hydrochloride (Ono Pharmaceutical Co., Ltd.) or a placebo (d-mannitol, 0.25 mg/kg) at a ratio of 1:1:1 before coronary CTA, and were treated with the bolus injection of the study drug after receiving a nitrate drug (nitroglycerin, 300–600 mg). First, 10 mL solution of the study drug (landiolol hydrochloride or placebo) was injected for 50 s, then 2–5 mL saline was injected for 10 s in all three groups.

### Coronary CT angiography

Coronary CTA was performed 4–7 min after the completion of the administration of the study drug. The CT equipment used included the SOMATOM Sensation Cardiac 64, the SOMATOM Sensation 64, and the SOMATOM Sensation 64-slice configuration (Siemens Healthcare, Forchheim, Germany). The CT equipment was operated with the X-ray tube voltage set at 120 kV, tube current at 770–850 mA, collimation at 32 rows × 0.6 mm, rotation speed of the X-ray tube at 0.33 s/rotation, helical pitch not more than 0.2, and the field of view at 200 mm. A nonionic contrast medium, iopamidol 370 mgI/mL (Iopamiron 370, Bayer, Berlin, German), was rapidly and intravenously injected at 3–4.5 mL/s using a 2-channel injector, followed by the infusion of 20–30 mL of saline using a bolus tracking system.

Image reconstruction was performed after applying the retrospective ECG-gated reconstruction method under optimal conditions at each study center. The slice thickness for reconstruction was set at 0.75 mm.

### Heart rate and safety assessment

The mean heart rate during CT scanning was measured using an ECG connected to the CT scanner, and was the average of heart rate throughout the scanning time (approximately 12 s). The heart rate (excluding the mean heart rate during CT scanning) and the blood pressure were monitored before the initiation of the study (baseline), upon admission to the CT imaging room, immediately before the administration of the nitrate drug, immediately before the administration of the study drug, every minute from 0 to 10 min after the completion of the administration of the study drug, and at 15 and 30 min after the completion of the administration of the study drug. The oxygen saturation (SpO_2_) was also monitored from the time of admission to the CT room until 30 min after the completion of the administration of the study drug. Additionally, a 12-lead ECG was performed, and laboratory values were assessed before the initiation of the study (baseline) and within 3 days after the completion of the administration of the study drug.

Physicians monitored adverse events from the initiation of the study, until the completion of the post-study safety observations (within 3 days after the coronary CTA). With regards to the definition of severity of an adverse event, investigators or authorized designees classified severity using the following classification criteria of side effect severity for drugs and related products (issued on 29 June 1992, by the Ministry of Welfare in Japan): mild, a minor adverse event; moderate, worse than a minor event, but not serious; severe, serious, where it can lead to death or a persistent dysfunction that will interfere with daily activity depending on a patient’s constitution or state.

### Image analysis

All CT data were sent to an independent central laboratory from each study site. Volume-rendered (VR) images and curved multi-planar reformation (MPR) images were reconstructed using an Aquarius NET Server workstation (Client PC networked with Aquarius NET Sever). The curved MPR images of each of 16 coronary segments in the American Heart Association (AHA) classification were assessed for coronary visualization according to 3 grades of motion artifacts by two radiodiagnostic specialists in consensus: Score 3, images with no motion artifact and that were diagnosable; Score 2, images with motion artifact(s), but that were diagnosable; and Score 1, images with motion artifact(s) not allowing diagnose. A score of either 3 or 2 was judged as being an assessable image. All of the assessable segments were then visually analyzed for the presence of stenosis (a luminal decrease ≥50 % was considered significant stenosis) by two other radiodiagnostic specialists who were required to reach a consensus. Coronary segments with a diameter <1.5 mm, heavily calcified segments, stented segments, segments distal to a total coronary occlusion, segments with poor contrast, and segments neighbored by a structure such as myocardial bridging were not included in the analysis. A patient’s imaging study was considered “valuable” if all of the coronary segments with a diameter >1.5 mm were assessable and free of stenosis, or if at least 1 area of stenosis had been detected by coronary CTA.

### Quantitative coronary angiography

Coronary angiography was conducted within 30 days of the coronary CTA. Experienced cardiologists performed the CAG using standard techniques, and standard projection planes were acquired. CAG images obtained from each study center were classified into 16 segments according to the AHA classification of coronary lesions. For each of the CAG images classified by segment, the Central CAG Stenosis Judgment Committee, consisting of two independent cardiologists, determined the coronary stenosis using quantitative coronary angiography (QCA). Coronary stenosis was evaluated using a cardiovascular analysis system (QCA-CMS, version 6.0; Medis), and a luminal decrease ≥50 % was considered significant stenosis.

### Statistical analysis

All of the CT and clinical data from each institute were analyzed statistically at the central laboratory. The sample size was set so that significant differences could be detected at a probability of ≥80 % when the difference in obtaining score of 2 or better for coronary visualization was ≥25 % between the placebo and 0.125-mg/kg of landiolol groups. Accordingly, the number of patients required was determined to be 54 per group, and the sample size was fixed at 55–60 patients per group, assuming that about 10 % of the required number of patients per group might discontinue study participation.

The patient background and coronary CTA conditions were tested for inter-group uniformity using the Chi square (χ^2^) test or the Kruskal–Wallis test. The assessable proportion and the performance of coronary diagnosis [sensitivity, specificity, positive predictive value (PPV), negative predictive value (NPV), and the proportion of correct classifications] in each patient and the vessel and segment in the 0.06 and 0.125-mg/kg landiolol hydrochloride groups were compared with those in the placebo group using the χ^2^ test as a post-hoc analysis. The changes in the heart rate, blood pressure, and SpO_2_ in the landiolol hydrochloride groups were compared to those in the placebo group using a *t* test. The incidence of adverse events was also examined using a *t* test.

## Results

A total of 183 patients were enrolled in the present study: 64 patients were randomized to the placebo group, 58 were randomized to the 0.06-mg/kg landiolol hydrochloride group (hereafter referred to as the “0.06-mg/kg” group), and 61 were randomized to the 0.125-mg/kg landiolol hydrochloride group (hereafter referred to as the “0.125-mg/kg” group).

All 183 participating patients were included in the safety analysis. Of these 183 patients, 20 were excluded from the efficacy analysis because of conflicts related to prior treatment therapy in two patients (protocol deviation for insufficient washout periods prior to study drug administration; 0.06-mg/kg group) and incomplete assessment data in 18 patients (protocol deviation for coronary CTA or missing data for coronary visualization; 6 patients each from the placebo group and the 0.06 and 0.125-mg/kg groups). Thus, 163 patients were included in the efficacy analysis: 58 patients from the placebo group, 50 from the 0.06-mg/kg group, and 55 from the 0.125-mg/kg group. Two patients in the 0.125-mg/kg group were excluded from the diagnostic performance analysis because of missing CAG data for all 4 arteries [right coronary artery (RCA), left main coronary artery (LMCA), left anterior descending (LAD), and left circumflex (LCX)]. Consequently, 161 patients were included in the diagnostic performance analysis.

### Baseline characteristics

The demographic factors and CT imaging conditions of the patients enrolled in the present study are summarized in Tables 1 and 2, respectively.

**Table 1:** Patient characteristics

Factor	Placebo (*n* = 64)	Landiolol hydrochloride, 0.06 mg/kg (*n* = 58)	Landiolol hydrochloride, 0.125 mg/kg (*n* = 61)	*P* value
Sex (male/female)	47/17 (73.4 %/26.6 %)	38/20 (65.5 %/34.5 %)	45/16 (73.8 %/26.2 %)	0.533^1)^
Age (years, mean ± SD)	64.2 ± 8.8	65.6 ± 9.4	65.5 ± 11.5	0.267^2)^
Height (cm, mean ± SD)	161.33 ± 8.61	159.11 ± 8.44	160.36 ± 10.02	0.236^2)^
Weight (kg, mean ± SD)	62.945 ± 10.433	61.232 ± 11.212	63.088 ± 11.219	0.715^2)^
No. of patients with symptoms	53 (82.8 %)	46 (79.3 %)	49 (80.3 %)	0.879^1)^
Baseline HR (beats/min, mean ± SD)	78.9 ± 9.2	79.4 ± 9.6	77.6 ± 10.0	0.341^2)^
Baseline SBP (mmHg, mean ± SD)	135.3 ± 21.7	138.6 ± 21.4	131.4 ± 18.9	0.173^2)^
Baseline DBP (mmHg, mean ± SD)	75.5 ± 11.5	78.5 ± 12.9	73.0 ± 11.5	0.049^2)^*
CAG-detected stenosis				
None	28 (43.8 %)	26 (45.6 %)	22 (36.7 %)	
Single branch	16 (25.0 %)	13 (22.8 %)	19 (31.7 %)	
2-Branch	11 (17.2 %)	12 (21.1 %)	10 (16.7 %)	
3-Branch	8 (12.5 %)	6 (10.5 %)	9 (15.0 %)	0.748^2)^
4-Branch	1 (1.6 %)			
Data missing		1	1	
Mean ± SD	1.0 ± 1.1	1.0 ± 1.1	1.1 ± 1.1	

*DBP* diastolic blood pressure, *HR* heart rate, *SBP* systolic blood pressure, *CAG* invasive coronary angiography

^1)^χ^2^-test

^2)^Kruskal–Wallis test

* *P* < 0.15

**Table 2:** Coronary CT imaging conditions

Factor	Placebo (*n* = 64)	Landiolol hydrochloride, 0.06 mg/kg (*n* = 58)	Landiolol hydrochloride, 0.125 mg/kg (*n* = 61)	*P* value
Time from completion of study drug administration until initiation of imaging (s, mean ± SD)	346.2 ± 95.7	336.4 ± 36.5	323.4 ± 47.5	0.237^2)^
CT imaging time (s, mean ± SD)	12.6 ± 1.6	12.6 ± 1.4	12.3 ± 1.5	0.474^2)^
Method of administration of contrast medium				
Rapid IV infusion at 3 mL/s	1 (1.6 %)	1 (1.7 %)	2 (3.3 %)	
Rapid IV infusion at 3.5 mL/s	6 (9.4 %)	7 (12.1 %)	3 (4.9 %)	0.596^1)^
Rapid IV infusion at 4 mL/s	25 (39.1 %)	21 (36.2 %)	21 (34.4 %)	
Rapid IV infusion at 4.5 mL/s	22 (34.4 %)	16 (27.6 %)	27 (44.3 %)	
Others	10 (15.6 %)	13 (22.4 %)	8 (13.1 %)	
Total dose of contrast medium and saline (mL, mean ± SD)	100.3 ± 10.1	98.3 ± 13.1	99.1 ± 11.3	0.655^2)^

^1)^χ^2^-test

^2)^Kruskal–Wallis test

The heart rates (mean ± SD) immediately before the administration of the study drug were 78.9 ± 9.2, 79.4 ± 9.6, and 77.6 ± 10.0 beats/min in the placebo, 0.06, and 0.125-mg/kg groups, respectively. At least one instance of coronary stenosis was detected in 56.3, 54.4, and 63.3 % of the patients in the placebo, 0.06, and 0.125-mg/kg groups, respectively. The CT imaging times were 12.6 ± 1.6, 12.6 ± 1.4, and 12.3 ± 1.5 s in the placebo, 0.06, and 0.125-mg/kg groups, respectively. The radiation dose for the coronary CTA was 11.9 ± 1.9 mSv for all the patients. No significant differences in the patient background characteristics were observed among the groups with the exception of the baseline systolic blood pressure.

### Heart rate evaluation

The mean heart rate at the time of coronary CTA was 73.7 ± 11.8, 67.6 ± 8.7, and 62.6 ± 7.8 beats/min in the placebo, 0.06, and 0.125-mg/kg groups, respectively; the values in the 0.06 and 0.125-mg/kg groups were significantly lower than that in the placebo group (*t* test: *P* = 0.003 and *P* < 0.001, respectively), and the value in the 0.125-mg/kg group was significantly lower than that in the 0.06-mg/kg group (*t* test: *P* = 0.002). In addition, the reductions in the heart rate, calculated as the percent change from the baseline to the measurement at the time of CTA, were −6.8 ± 10.4 %, −14.5 ± 8.3 %, and −19.2 ± 9.3 % in the placebo, 0.06, and 0.125-mg/kg groups, respectively. The reduction in heart rate was significantly greater in both the 0.06 and the 0.125-mg/kg groups than in the placebo group (*t* test: both *P* < 0.001), and the rate in the 0.125-mg/kg group was significantly lower than that in the 0.06-mg/kg group (*t* test: *P* = 0.007).

**Fig. 1:**
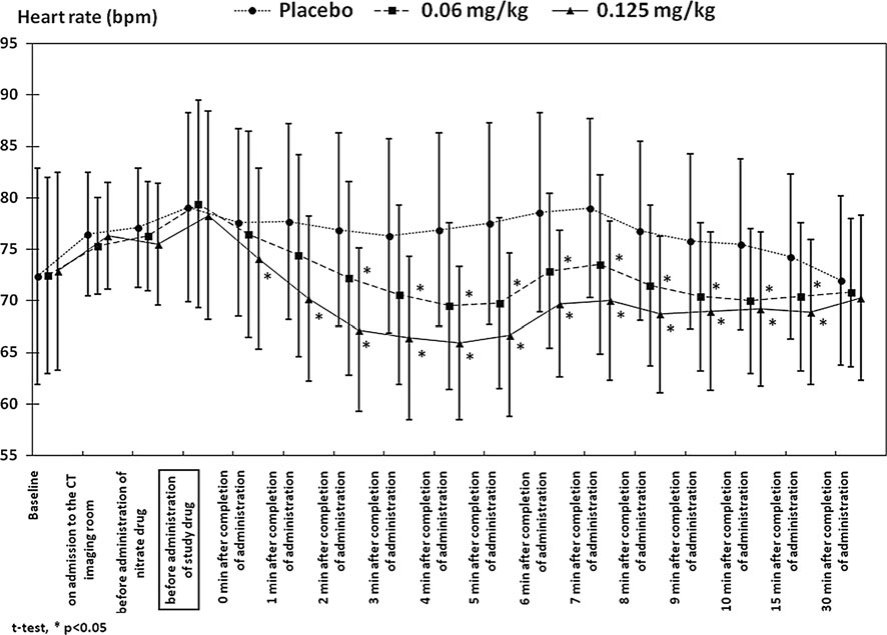
Changes in heart rate during and after CT examination

As shown in Fig. 1, the rapid reduction in the heart rate started immediately after the administration of the study drug in both the 0.06 and the 0.125-mg/kg groups, and became significant at 2–15 min after the completion of the administration in the 0.06-mg/kg group, and at 0–15 min in the 0.125-mg/kg group. However, 30 min after the completion of the administration of the study drug, the heart rate was not significantly lower in either the 0.06 or the 0.125-mg/kg groups, compared to the placebo group.

### Safety

**Fig. 2:**
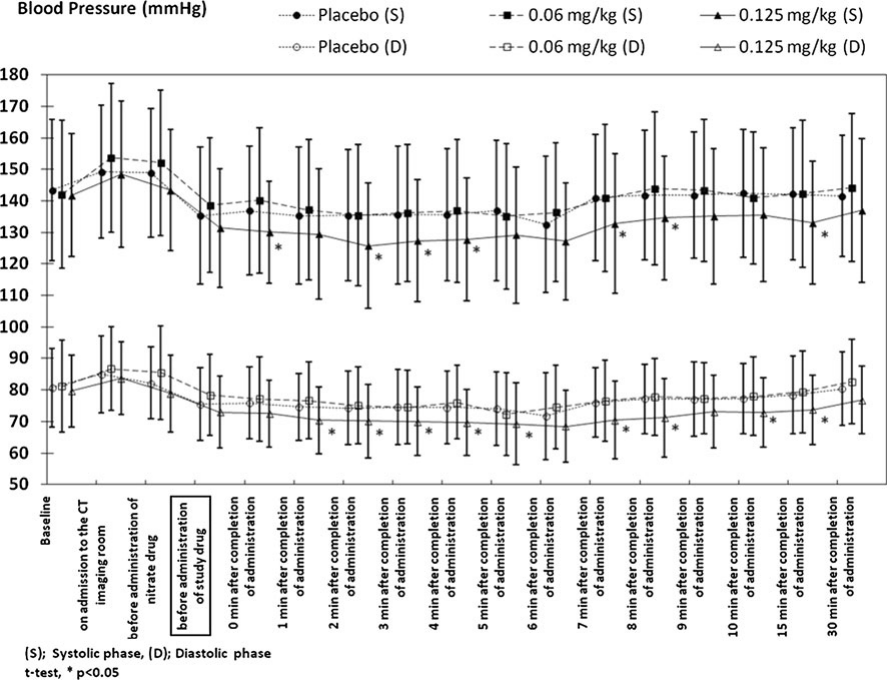
Changes in systolic and diastolic blood pressure during and after CT examination

**Fig. 3:**
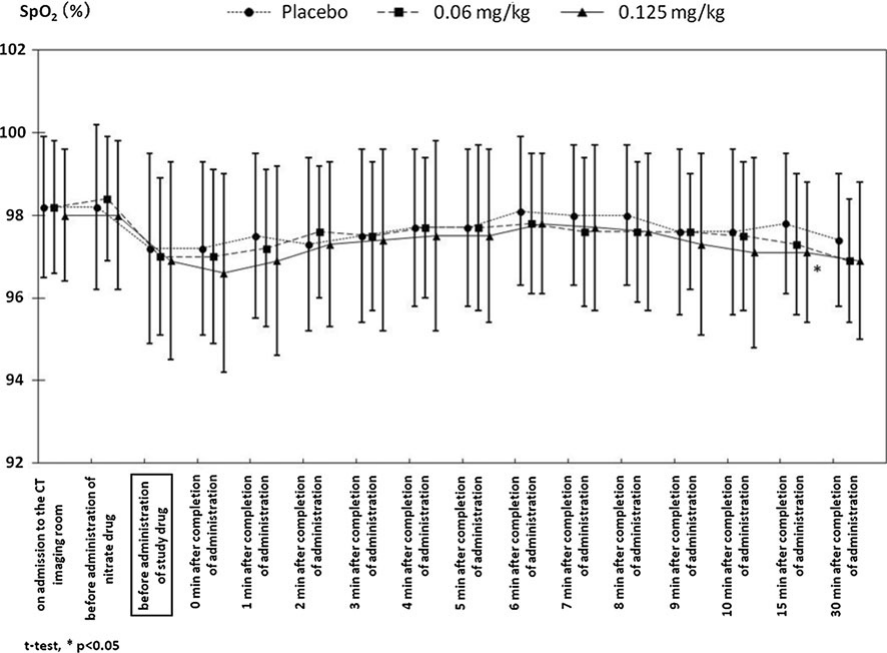
Changes in SpO_2_ during and after CT examination

The change in the blood pressure is shown in Fig. 2. A reduction in blood pressure was noted in the 0.06 and the 0.125-mg/kg groups, but similar to the heart rate, the blood pressure was no longer significantly lower than that in the placebo group at 30 min after the completion of the administration of the study drug. In addition, as shown in Fig. 3, the reduction in SpO_2_ was not significantly lower in the 0.06 and the 0.125-mg/kg groups, compared with that in the placebo group, except at 15 min after the administration of the study drug in the 0.125-mg/kg group.

As shown in Table 3, no adverse events occurred at an incidence of 5 % or greater in any of the treatment groups. An increase in creatine phosphokinase (CPK) and a reduction in blood pressure were each observed in two subjects in the 0.125-mg/kg group. Other adverse events were each observed in only one subject in one of the groups. The incidences of all adverse events (any unfavorable and unintended sign, symptom, or disease temporally associated with the use of study, whether or not considered related to study drug) were 9.4 (6/64 patients), 10.3 (6/58 patients), and 14.8 (9/61 patients) in the placebo, 0.06, and 0.125-mg/kg groups, respectively, and the incidences of all adverse reactions (any adverse events whose relationship to study drug cannot be ruled out) were similar: 6.3 (4/64 patients), 3.4 (2/58 patients), and 9.8 (6/61 patients), respectively, with no significant differences among the three groups. No deaths were observed during the study period, and all of the adverse events were mild except for one case of a severe acute myocardial infarction. The severe acute myocardial infarction was observed in one patient in the 0.06-mg/kg group. The infarction occurred on day 2 after the administration of the study drug, and was considered to be attributable to a complication (angina pectoris) in this patient. The attending physician judged the case as being “unrelated to the study drug.” Other significant adverse events included 2 cases of a mild decrease in blood pressure in 2 patients in the 0.125-mg/kg group. In one case, the blood pressure just prior to the administration of study drug was 102/37 mmHg, with a decrease to 79/30 mmHg observed at 6 min after the initiation of the administration of the study drug, but a pressure of 125/55 mmHg was observed 3 min later. The patient’s hypotension eventually resolved without requiring treatment. In the other case, the blood pressure just prior to the administration of study drug was 122/68 mmHg, with a decrease to 95/62 mmHg observed at 3 min after the initiation of the administration of the study drug. Increase to a pressure of 115/61 mmHg was observed 7 min later, and these changes also eventually resolved without requiring treatment and thus posed no clinical concern. In neither of these cases did the effect of the β-receptor blocker persist after the completion of the study. In addition, the measurements of ECG parameters including the RR interval, PQ interval, QRS duration, QT interval, QTc (Bazett formula: QTc = QT/RR^0.5^), and ST segment elevation, as well as differences from the baseline measurements, revealed no significant changes in the 0.06 or 0.125-mg/kg group compared with the values in the placebo group.

**Table 3:** Incidence of adverse events

Severity	Placebo									0.06-mg/kg group								
	Mild		Moderate		Severe		Total		N	Mild		Moderate		Severe		Total		N
	n	(%)	n	(%)	n	(%)	n	(%)		n	(%)	n	(%)	n	(%)	n	(%)	
Total	6	(9.4)					6	(9.4 )	64	5	(8.6)			1	(1.7)	6	(10.3)	58
Anemia																		
Iron deficiency anemia	1	(1.6)					1	(1.6)	64									
Acute cardiac infarction														1	(1.7)	1	(1.7)	58
Chest discomfort	***1***	***(1.6)***					***1***	***(1.6)***	64									
Increase in ALT	1	(1.6)					1	(1.6)	63^a^									
Increase in AST	1	(1.6)					1	(1.6)	63^a^									
Decrease in serum albumin level	1	(1.6)					1	(1.6)	63^a^									
Increase in CPK	***1***	***(1.6)***					***1***	***(1.6)***	63^a^	1	(1.7)					1	(1.7)	58
Increase in creatinine										1	(1.7)					1	(1.7)	58
Increase in serum potassium level	***1***	***(1.6)***					***1***	***(1.6)***	63^a^	*** 1***	***(1.7)***					***1***	***(1.7)***	58
Hypotension																		
Increase in blood urea nitrogen										1	(1.7)					1	(1.7)	58
Decrease in hematocrit	1	(1.6)					1	(1.6)	63^a^									
Decrease in hemoglobin	1	(1.6)					1	(1.6)	63^a^									
Decrease in total protein	1	(1.6)					1	(1.6)	62^a^									
Decrease in red cell count	1	(1.6)					1	(1.6)	63^a^									
Increase in white cell count	***1***	***(1.6)***					***1***	***(1.6)***	63^a^									
Increase in alkaline phosphatase										*** 1***	***(1.8)***					***1***	***(1.8)***	57^a^
Headache																		
Nasal congestion																		
Sneezing																		
Anthema										1	(1.7)					1	(1.7)	58
Hives										*** 1***	***(1.7)***					***1***	***(1.7)***	58

Severity	0.125-mg/kg group								
	Mild		Moderate		Severe		Total		N
	n	(%)	n	(%)	n	(%)	n	(%)	
Total	9	(14.8)					9	(14.8)	61
Anemia	1	(1.6)					1	(1.6)	61
Iron deficiency anemia									
Acute cardiac infarction									
Chest discomfort									
Increase in ALT									
Increase in AST									
Decrease in serum albumin level	***1***	***(1.6)***					***1***	***(1.6)***	61
Increase in CPK	2	(3.3)					2	(3.3)	61
Increase in creatinine	***1***	***(1.6)***					***1***	***(1.6)***	61
Increase in serum potassium level									
Hypotension	***2***	***(3.3)***					***2***	***(3.3)***	61
Increase in blood urea nitrogen									
Decrease in hematocrit	1	(1.6)					1	(1.6)	61
Decrease in hemoglobin	1	(1.6)					1	(1.6)	61
Decrease in total protein	***1***	***(1.6)***					***1***	***(1.6)***	61
Decrease in red cell count	1	(1.6)					1	(1.6)	61
Increase in white cell count	***1***	***(1.6)***					***1***	***(1.6)***	61
Increase in alkaline phosphatase									
Headache	1	(1.6)					1	(1.6)	61
Nasal congestion	***1***	***(1.6)***					***1***	***(1.6)***	61
Sneezing	***1***	***(1.6)***					***1***	***(1.6)***	61
Anthema	***1***	***(1.6)***					***1***	***(1.6)***	61
Hives									

Bold Italic, classified as events related to study medication

^a^ “N” indicates total number of subjects with each laboratory data. One or two laboratory values were missing, even though their laboratory tests were examined

### Diagnostic performance

**Fig. 4:**
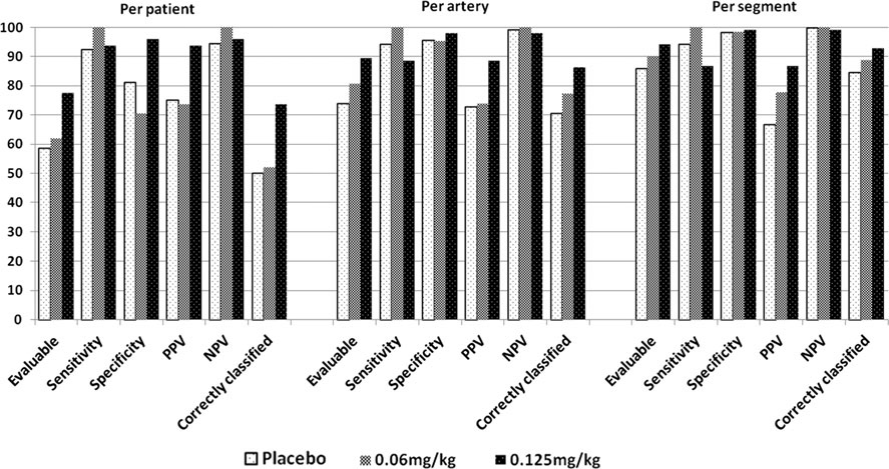
Diagnostic performance compared on a per-patient, per-artery, and per-segment basis

Table 4 and Fig. 4 present the diagnostic performance results per patient, per vessel, and per segment.

**Table 4:** Diagnostic performance on coronary CT angiography

		Placebo			0.06-mg/kg group				0.125-mg/kg group			
		n/total	Proportion (%)	95 % CI	n/total	Proportion (%)	95 % CI	*P* value	n/total	Proportion (%)	95 % CI	*P* value^1)^
Per patient	Assessable	34/58	(58.6)	(44.9, 71.4)	31/50	(62.0)	(47.2, 75.3)	0.721	41/53	(77.4)	(63.8, 87.7)	0.035*
	Sensitivity	12/13	(92.3)	(64.0, 99.8)	14/14	(100.0)	(76.8, 100.0)	0.290	15/16	(93.8)	(69.8, 99.8)	0.879
	Specificity	17/21	(81.0)	(58.1, 94.6)	12/17	(70.6)	(44.0, 89.7)	0.455	24/25	(96.0)	(79.6, 99.9)	0.102
	PPV	12/16	(75.0)	(47.6, 92.7)	14/19	(73.7)	(48.8, 90.9)	0.929	15/16	(93.8)	(69.8, 99.8)	0.144
	NPV	17/18	(94.4)	(72.7, 99.9)	12/12	(100.0)	(73.5, 100.0)	0.406	24/25	(96.0)	(79.6, 99.9)	0.811
	Correctly Classified	29/58	(50.0)	(36.6, 63.4)	26/50	(52.0)	(37.4, 66.3)	0.836	39/53	(73.6)	(59.7, 84.7)	0.011*
Per artery	Assessable	153/207	(73.9)	(67.4, 79.8)	145/180	(80.6)	(74.0, 86.1)	0.121	168/188	(89.4)	(84.0, 93.4)	<0.001*
	Sensitivity	16/17	(94.1)	(71.3, 99.9)	17/17	(100.0)	(80.5, 100.0)	0.310	23/26	(88.5)	(69.8, 97.6)	0.532
	Specificity	130/136	(95.6)	(90.6, 98.4)	122/128	(95.3)	(90.1, 98.3)	0.914	139/142	(97.9)	(94.0, 99.6)	0.279
	PPV	16/22	(72.7)	(49.8, 89.3)	17/23	(73.9)	(51.6, 89.8)	0.928	23/26	(88.5)	(69.8, 97.6)	0.164
	NPV	130/131	(99.2)	(95.8, 100.0)	122/122	(100.0)	(97.0, 100.0)	0.334	139/142	(97.9)	(94.0, 99.6)	0.354
	Correctly Classified	146/207	(70.5)	(63.8, 76.6)	139/180	(77.2)	(70.4, 83.1)	0.136	162/188	(86.2)	(80.4, 90.8)	<0.001*
Per segment	Assessable	500/582	(85.9)	(82.8, 88.6)	420/466	(90.1)	(87.1, 92.7)	0.038*	517/549	(94.2)	(91.9, 96.0)	<0.001*
	Sensitivity	16/17	(94.1)	(71.3, 99.9)	21/21	(100.0)	(83.9, 100.0)	0.260	26/30	(86.7)	(69.3, 96.2)	0.426
	Specificity	475/483	(98.3)	(96.8, 99.3)	393/399	(98.5)	(96.8, 99.4)	0.857	483/487	(99.2)	(97.9, 99.8)	0.239
	PPV	16/24	(66.7)	(44.7, 84.4)	21/27	(77.8)	(57.7, 91.4)	0.375	26/30	(86.7)	(69.3, 96.2)	0.079
	NPV	475/476	(99.8)	(98.8, 100.0)	393/393	(100.0)	(99.1, 100.0)	0.363	483/487	(99.2)	(97.9, 99.8)	0.187
	Diagnostic accuracy	491/582	(84.4)	(81.2, 87.2)	414/466	(88.8)	(85.6, 91.6)	0.036*	509/549	(92.7)	(90.2, 94.7)	<0.001*

*NPV* negative predictive value, *PPV* positive predictive value

^1)^χ^2^-test

* *P* < 0.05

#### Per-patient analysis

Overall, 161 patients (58, 50, and 53 patients in each group) were included. The proportions of assessable patients were 58.6 % [34/58 patients; 95 % confidence interval (CI) 44.9–71.4 %], 62.0 % (31/50 patients; 95 % CI 47.2–75.3 %), and 77.4 % (41/53 patients; 95 % CI 63.8–87.7 %) in the placebo, 0.06, and 0.125-mg/kg groups, respectively, and the value in the 0.125-mg group was significantly higher than that in the placebo group (*P* = 0.035). However, no significant difference was observed between the 0.06-mg/kg group and the placebo group (*P* = 0.721). Regarding the coronary diagnostic performance in the placebo, 0.06, and 0.125-mg/kg groups, the sensitivities were 92.3, 100.0, and 93.8 %, respectively; the specificities were 81.0, 70.6, and 96.0 %, respectively; the positive predictive values (PPVs) were 75.0, 73.7, and 93.8 %, respectively; and the negative predictive values (NPVs) were 94.4, 100.0, and 96.0 %, respectively. The proportions of correct classification were 50.0 % (29/58 patients; 95 % CI 36.6–63.4 %), 52.0 % (26/50 patients; 95 % CI 37.4–66.3 %), and 73.6 % (39/53 patients; 95 % CI 59.7–84.7 %), respectively. The proportion of correct classification was significantly higher in the 0.125-mg/kg group than in the placebo group (*P* = 0.011). However, no difference was observed between the 0.06-mg/kg group and the placebo group (*P* = 0.836).

#### Per-artery analysis

Of the 644 arteries that were examined in total (i.e., RCA, LM, LAD, and LCX in 161 patients), 575 arteries were included. In the placebo, 0.06, and 0.125-mg/kg groups, a total of 23, 15, and 19 arteries, respectively, were excluded because of severe calcification. Twelve other arteries were also excluded because of the placement of stents (6 arteries), a diameter <1.5 mm (4 arteries), and CT data with poor contrast (2 arteries). The assessable proportions were 73.9 % (153/207 arteries; 95 % CI 67.4–79.8 %), 80.6 % (145/180 arteries; 95 % CI 74.0–86.1 %), and 89.4 % (168/188 arteries; 95 % CI 84.0–93.4 %) in the placebo, 0.06, and 0.125-mg/kg groups, respectively. The proportion in the 0.125-mg/kg group was significantly higher than that in the placebo group (*P* < 0.001), however no difference was observed between the 0.06-mg/kg group and the placebo group (*P* = 0.121). Regarding the coronary diagnostic performances in the placebo, 0.06, and 0.125-mg/kg groups, the sensitivities were 94.1, 100.0, and 88.5 %, respectively; the specificities were 95.6, 95.3, and 97.9 %, respectively; the PPVs were 72.7, 73.9, and 88.5 %, respectively; and the NPVs were 99.2, 100.0, and 97.9 %, respectively. The proportions of correct classification were 70.5 % (146/207 arteries; 95 % CI 63.8–76.6 %), 77.2 % (139/180 arteries; 95 % CI 70.4–83.1 %), and 86.2 % (162/188 arteries; 95 % CI 80.4–90.8 %), respectively. The proportion of correct classification was significantly higher in the 0.125-mg/kg group than in the placebo group (*P* < 0.001), however again no difference was observed between the 0.06-mg/kg group and the placebo group (*P* = 0.136).

#### Per-segment analysis

Overall, 1,597 segments were included in the analysis. Most of the excluded segments were excluded because of severe calcification (487 segments in total: 172 in the placebo group, 165 in the 0.06-mg/kg group, and 152 in the 0.125-mg/kg group). Additionally, 344 segments (118 in the placebo group, 122 in the 0.06-mg/kg group, and 104 in the 0.125-mg/kg group) were excluded because they had a diameter <1.5 mm, and 75, 17, and 8 segments were excluded because of the placement of stents, the presence of a myocardial bridge, and poorly visualized CT data, respectively. The assessable proportions were 85.9 % (500/582 segments; 95 % CI 82.8–88.6 %), 90.1 % (420/466 segments; 95 % CI 87.1–92.7 %), and 94.2 % (517/549 segments; 95 % CI 91.9–96.0 %) in the placebo, 0.06, and 0.125-mg/kg groups, respectively, and the values in the 0.06 and 0.125-mg/kg groups were significantly higher than that in the placebo group (*P* = 0.038 and *P* < 0.001, respectively). Regarding the coronary diagnostic performances in the placebo, 0.06, and 0.125-mg/kg groups, the sensitivities were 94.1, 100.0, and 86.7 %, respectively; the specificities were 98.3, 98.5, and 99.2 %, respectively; the PPVs were 66.7, 77.8, and 86.7 %, respectively; and the NPVs were 99.8, 100.0, and 99.2, respectively. The proportions of correct classification were 84.4 % (491/582 segments; 95 % CI 81.2–87.2 %), 88.8 % (414/466 segments; 95 % CI 85.6–91.6 %), and 92.7 % (509/549 segments; 95 % CI 90.2–94.7 %), respectively. The proportions of correct classification were significantly higher in the 0.06 and 0.125-mg/kg groups than in the placebo group (*P* = 0.036 and *P* < 0.001, respectively).

## Discussion

Very few studies have reported the clinical use of the β_1_-receptor selective blocker landiolol hydrochloride [19, 20]. Isobe et al. [19] reported the usefulness of the continuous injection of landiolol hydrochloride. However, the continuous injection method is complicated. Instead of a continuous injection, Osawa et al. reported the usefulness of a more practical bolus injection method for administering landiolol hydrochloride at a dose of 0.125 mg/kg. However, in their study, the patients arrived at the hospital 1 h before the scheduled scanning time and initially received oral β-receptor blockers. When the oral β-receptor blocker was not effective, landiolol hydrochloride was administered as additional pretreatment. Thus, they evaluated the usefulness of landiolol hydrochloride used in combination with an oral β-receptor blocker. This manner of use did not shorten the examination time. In the present study, we evaluated the usefulness and safety of a bolus injection of landiolol hydrochloride at a dose of 0.06 or 0.125 mg/kg using a placebo-controlled study design without the prior use of oral β-receptor blockers.

In this study, the reduction in the heart rate of the 0.125-mg/kg group was significantly higher than that in both the 0.06-mg/kg group and the placebo group (both *P* < 0.01). The injection of the study drug rapidly reduced the heart rate soon after administration, and the reduction in the heart rate was significant immediately after administration in the 0.125-mg/kg group, and at 2 min after administration in the 0.06-mg/kg group. However, at 30 min after the administration, the heart rate in these two groups did not differ significantly from that in the placebo group. This data demonstrated that landiolol hydrochloride can be administered immediately before coronary CTA, and that the β-blocking effect is not prolonged after the coronary CTA. When oral β-receptor blockers are used, patients must visit the hospital 1–2 h before the coronary CTA to take the β-blocker, and must allow their heart rate to be monitored to determine whether it meets the conditions required for CT imaging. The administration of 0.125 mg/kg of landiolol hydrochloride shortened the examination time, and increased the efficiency of coronary CTA.

Regarding the safety of landiolol hydrochloride, no adverse events or reactions were seen at an incidence of 5 % or greater. While β_1_ blocker-induced bradyarrhythmia and hypotension, and β_2_ blocker-induced bronchoconstriction and peripheral circulatory disorders are known adverse reactions to nonselective β-blockers, the principal adverse reactions of landiolol hydrochloride are likely to be only bradyarrhythmia and hypotension, given the high selectivity of this drug to β_1_ receptors (β_1_/β_2_: 251/1) [21]. In the present study, none of the patients developed bradyarrhythmia, and the mild decreases in blood pressure observed in 2 patients treated with 0.125 mg/kg of landiolol hydrochloride rapidly resolved without any treatment, and posed no clinical concerns.

In previous studies evaluating the diagnostic performance of coronary CTA with a Siemens 64-row CT imaging system, the heart rate was usually regulated at lower than 65 beats/min through the administration of an oral β-blocker. Only a few studies have evaluated the diagnostic performance in patients with a high heart rate, such as in the placebo group in our study. In the present placebo-controlled study, only patients who exhibited a high heart rate of 70–90 beats/min prior to undergoing a coronary CTA were included. To our knowledge, only one study reported the diagnostic performance of patients with no heart rate control, in which segments with a diameter of <1.5 mm were excluded, similar to our study [22]. In this previous study, the proportions of correct classification per patient, vessel, and segment in the “no” heart rate control group were 57, 77, and 80 %, respectively, which were similar to the results obtained in our placebo group of 50, 70.5, and 84.4 %, respectively. Also, in this previous study, the proportions of correct classification per patient, vessel, and segment improved in the heart rate control group to 78, 86, and 91 %, respectively, with the use of a Siemens 64-row CT scanner, similar to the results obtained in our 0.125-mg/kg group (73.6, 86.2, and 92.7 %, respectively). The proportions of correct classification per patient and per vessel were significantly higher in the 0.125-mg/kg group than in the placebo group, but not in the 0.06-mg/kg group. Additionally, the proportion of correct classification per segment was higher in both the 0.06 and the 0.125-mg/kg groups than in the placebo group.

The present study has several limitations. First, among the coronary CTA scanners commonly used in clinical practice, only the Siemens 64-row CT scanner was used in the present study. Further studies using CT scanners manufactured by other companies are anticipated. Second, as part of the eligibility criteria in the present study, patients with a heart rate that was over 90 beats/min before the coronary CTA procedure, those who were presumed to have developed an arrhythmia at the time of the coronary CTA, and those who had undergone coronary artery bypass surgery were excluded. Thus, the usefulness of landiolol hydrochloride in these patients should be further evaluated. Third, we did not directly compare the safety and efficacy between this drug and oral beta-blockers, which is expected to be evaluated in further studies. Finally, we measured only the averaged heart rate during the scanning time, and did not measure the heart rate variability, which was reported to be a determinant of image quality on 64-section CT in one study [23]. Isobe et al. [19] demonstrated that heart rate variability was significantly reduced during CT acquisition with administration of landiolol hydrochloride beforehand, although the difference of heart rate variability was only 1.8 bpm on average (4.1 ± 1.8 vs. 2.3 ± 1.4 bpm). We expect to clarify whether decreasing of heart rate variability by using landiolol hydrochloride can affect any improvement of image quality in a future study.

In conclusion, intravenous injection of landiolol hydrochloride appears to be useful for significantly reducing the heart rate during the period necessary for the coronary CTA and significantly improves the diagnostic performance, compared with a placebo group, with no significant adverse events or reactions. The clinically recommended dose of landiolol hydrochloride based on this study is 0.125 mg/kg.

## Appendix

A complete list of the members of the landiolol hydrochloride Study Group follows.

Trial members: All authors and Teruhito Mochizuki, Hiroshi Higashino, Masahiro Higashi, Tetsuya Fukuda, Teruhito Kido, Yoshiro Hori, Yoshihiro Morino, and Naoki Masuda.

Principal investigators: Yuji Ikari (Isehara-city, Kanagawa), Hiroshi Inoue (Toyama-city, Toyama), Norio Urabe (Ako-city, Hyogo), Mariko Ehara (Toyohashi-city, Aichi), Hideyuki Sakai (Ota-ku, Tokyo), Kennei Shimada (Nishi-ku, Osaka), Michihisa Jogasaki (Kagoshima-city, Kagoshima), Shoji Suzuki (Kasama-city, Ibaraki), Masahiro Tamashiro (Tomigusuku-city, Okinawa), Yawara Nijima (Takasaki-city, Gunma), Kazuhiro Hara (Chiyoda-ku, Tokyo), Yoshikazu Hiasa (Komatsujima-city, Tokushima), Yuji Hiraoka (Kyoto-city, Kyoto), Toshikazu Funazaki (Kawaguchi-city, Saitama), Hiroshi Maeda (Inushima-gun, Ibaraki), Masunori Matsuzaki (Ube-city, Yamaguchi), Takashi Murou (Abeno-ku, Osaka), and Akira Yamashina (Shinjuku-ku, Tokyo).
